# Using Machine Learning of Online Expression to Explain Recovery Trajectories: Content Analytic Approach to Studying a Substance Use Disorder Forum

**DOI:** 10.2196/45589

**Published:** 2023-08-22

**Authors:** Ellie Fan Yang, Rachel Kornfield, Yan Liu, Ming-Yuan Chih, Prathusha Sarma, David Gustafson, John Curtin, Dhavan Shah

**Affiliations:** 1 School of Communication and Mass Media Northwest Missouri State University Maryville, MO United States; 2 Feinberg School of Medicine, Northwestern University Chicago, IL United States; 3 School of Journalism and Communication, Shanghai University Shanghai China; 4 College of Health Science, University of Kentucky Lexington, KY United States; 5 Apple Inc Cupertino, CA United States; 6 Department of Psychology, University of Wisconsin-Madison Madison, WI United States

**Keywords:** supervised machine learning, online peer support forum, expression effects, content analysis, substance use disorder, mobile phone

## Abstract

**Background:**

Smartphone-based apps are increasingly used to prevent relapse among those with substance use disorders (SUDs). These systems collect a wealth of data from participants, including the content of messages exchanged in peer-to-peer support forums. How individuals self-disclose and exchange social support in these forums may provide insight into their recovery course, but a manual review of a large corpus of text by human coders is inefficient.

**Objective:**

The study sought to evaluate the feasibility of applying supervised machine learning (ML) to perform large-scale content analysis of an online peer-to-peer discussion forum. Machine-coded data were also used to understand how communication styles relate to writers’ substance use and well-being outcomes.

**Methods:**

Data were collected from a smartphone app that connects patients with SUDs to online peer support via a discussion forum. Overall, 268 adult patients with SUD diagnoses were recruited from 3 federally qualified health centers in the United States beginning in 2014. Two waves of survey data were collected to measure demographic characteristics and study outcomes: at baseline (before accessing the app) and after 6 months of using the app. Messages were downloaded from the peer-to-peer forum and subjected to manual content analysis. These data were used to train supervised ML algorithms using features extracted from the Linguistic Inquiry and Word Count (LIWC) system to automatically identify the types of expression relevant to peer-to-peer support. Regression analyses examined how each expression type was associated with recovery outcomes.

**Results:**

Our manual content analysis identified 7 expression types relevant to the recovery process (emotional support, informational support, negative affect, change talk, insightful disclosure, gratitude, and universality disclosure). Over 6 months of app use, 86.2% (231/268) of participants posted on the app’s support forum. Of these participants, 93.5% (216/231) posted at least 1 message in the content categories of interest, generating 10,503 messages. Supervised ML algorithms were trained on the hand-coded data, achieving *F*_1_-scores ranging from 0.57 to 0.85. Regression analyses revealed that a greater proportion of the messages giving emotional support to peers was related to reduced substance use. For self-disclosure, a greater proportion of the messages expressing universality was related to improved quality of life, whereas a greater proportion of the negative affect expressions was negatively related to quality of life and mood.

**Conclusions:**

This study highlights a method of natural language processing with potential to provide real-time insights into peer-to-peer communication dynamics. First, we found that our ML approach allowed for large-scale content coding while retaining moderate-to-high levels of accuracy. Second, individuals’ expression styles were associated with recovery outcomes. The expression types of emotional support, universality disclosure, and negative affect were significantly related to recovery outcomes, and attending to these dynamics may be important for appropriate intervention.

## Introduction

### Background

An estimated 19.7 million Americans have a substance use disorder (SUD) in their lifetime, yet few receive treatment [1]. Furthermore, recovery outcomes are poor even among those receiving treatment, with more than two‐thirds returning to the use of substances within months of leaving treatment [2,3]. Given that SUDs are serious and stigmatized conditions, bolstering online peer-to-peer communication can play a crucial role in recovery, offering an accessible and comfortable social context within which to discuss challenges, give and receive social support, and build a positive social identity around recovery [4,5]. Smartphone-based support apps are increasingly used to sustain peer support for recovery and prevent relapse [6,7]. These apps can also unobtrusively collect rich data about participants’ communication with peers, including the content of messages exchanged and surrounding metadata.

The idea of connecting individuals’ patterns of expression to their recovery outcomes predates the emergence of app-based communication; for example, prior work has applied content analysis methods to therapists’ interactions with patients in recovery for SUDs, finding that certain patient utterances, such as relaying motivations for change, precede reductions in substance use [8]. Through unobtrusively collecting a wealth of text data, online peer-to-peer support forums now offer a potential avenue to clarify the relationship between communication and recovery. Simultaneously, recent developments in natural language processing and machine learning (ML) provide new opportunities to take advantage of the huge volume of unstructured text in online support forums and other health care applications [9-16]. However, few studies have merged online text with survey-based measures of patients’ health and behaviors to understand how communication styles within a peer-to-peer forum relate to SUD recovery outcomes [17].

This study examined communication styles within an app-based peer-to-peer forum for primary care patients with SUDs. In the next subsection, we review the forms of communication indicated in prior literature as potentially relevant in peer-to-peer communication and that could affect patients’ recovery course in the context of SUDs.

### Types of Expression in Online Support Forums

It remains unclear what types of peer-to-peer communication, especially what types of expression, are key to activating recovery benefits*.* Social support is a dominant expression type of online recovery support forums, but individuals also frequently share their thoughts, feelings, and experiences with others through these forums (ie, *self-disclosure*). Self-disclosure may play an important role in recovery by soliciting social support [18]; allowing individuals to work through, and understand, their challenges [19]; or deepening relationships with others [20]. Thus, expressing both social support and self-disclosure is likely to play a role in helping individuals to adjust to recovery, sustain motivation, and enhance their sense of connectedness and general well-being. In the following paragraphs, we summarize what the literature suggests about how specific forms of social support and self-disclosure may factor in health and well-being outcomes in recovery.

Across previous research, emotional support and informational support are the 2 most studied types of communication in peer-to-peer contexts [21,22]. Emotional support refers to messages that help meet recipients’ emotional needs, such as by enhancing feelings of being understood, respected, or cared for [18]. It is one of the most common forms of communication across numerous types of online support forums (eg, recovery, cancer, and grief [23]). Although much social support literature focuses on benefits that come from *receiving* emotional support, evidence suggests that *providing* emotional support can also be beneficial because it may help the providers to build relationships and develop a sense of purpose [20,24]; for example, expressing emotional support had a salutary effect on mental well-being among patients with cancer with high communication competence [25] and led to effective coping strategies [26]. Accordingly, giving emotional support is likely to be positively related to recovery outcomes.

Another type of social support communication in the context of SUDs is informational support, which refers to suggestions or information aimed at helping recipients to manage their recovery [27]. This could involve furnishing information about treatment options, coping skills, or solutions to day-to-day problems [27,28]. In addition to helping the receiver to navigate recovery, some evidence suggests that giving informational support may foster self-reflection and reappraisal of one’s own problems [29]. Consistent with this, patients with alcohol use disorder had reduced relapse likelihood when they provided informational support [28,30]. However, giving informational support can be challenging because support providers must *match* their support to the recipient’s particular challenge [21,31]. Nonetheless, we expect that providing peer-to-peer informational support will result in improved recovery outcomes.

Beyond social support, self-disclosure, which is defined as “the act of revealing personal information to others” [32], such as feelings, thoughts, and experiences [33], also plays an important role in support forums. A functional perspective suggests that self-disclosure allows communicators to meet diverse strategic goals, depending on the audiences reached and the disclosure context [34,35]. Self-disclosure in a supportive social context may help individuals to process experiences and emotions for effective coping [36,37] as well as elicit reciprocal disclosure [38], with multiple forms of self-disclosure being potentially relevant to recovery.

The first common type of self-disclosure is negative affect expression [18]. This reflects that stress, depression, and frustration often accompany illness [39]. Consistent with the *expressive writing* paradigm, some experimental work suggests that expressing difficult feelings allows individuals to make sense of these experiences and find solutions [40,41]. In social contexts such as peer-to-peer forums, expressing one’s challenges may also lead to receiving more social support [18,42]. However, other studies found that negative emotional disclosures do not always generate peer responsiveness [43,44]. Expressing negative affect can also signal that an individual is facing a more challenging recovery course, preceding relapse [45,46]. Therefore, the relationship between negative affect disclosure and recovery outcomes is unclear.

Another well-studied form of self-disclosure involves generating commitments to behavior change. Within motivational interviewing, “change talk” refers to “self-expressed language that is an argument for change” [47], including conveying the ability, need, desire, or reasons to shift behaviors; commitment to change; or referencing actions linked to behavioral adjustments. Putting one’s argument for change into their language use may increase awareness about discrepancies between one’s goals and current behaviors, motivating action [48]. As such, therapy often aims to create contexts for patients to engage in change talk [49,50]. In SUDs, such talk has been linked to persistence in treatment and reduced substance use [51], reduced alcohol-related negative consequences [52], and increased abstinence from drug use [49]. However, change talk has predominantly been examined in face-to-face therapist-client sessions, leaving it unclear to what extent a shift of language use occurs in online peer-to-peer forums and whether spontaneous peer-to-peer change talk relates to recovery outcomes.

A third type of self-disclosure involves the expression of insights. Reflecting on, writing about, and sharing one’s experiences can facilitate cognitive processing that ultimately fosters an awareness of triggering factors and that may potentially support one in taking new actions to cope with, and address, stressors [19,53]. Insightful disclosure is related to individuals’ adjustment to recovery in various health contexts, including recovering from breast cancer [53] and anorexia [54], suggesting that it may be positively related to an individual’s recovery outcomes.

Additional forms of self-disclosure capture how the communicator reflects on feelings about others within the support group itself; for example, gratitude expression, a common feature of peer-to-peer SUDs forums [18], involves individuals sharing their feelings of thankfulness or appreciation toward those in their life [55]. Some health interventions have sought to promote gratitude expression [56] in the belief that it can enhance recovery outcomes.

Similarly, given that peer support brings together individuals with a common condition or identity, a core function of online peer support groups can be promoting a feeling of shared experience [57,58]. This feeling of “universality” describes “the idea that people have the same experiences or report similar experiences, circumstances, or feelings” [59]. Although less studied in online peer support, expressions of universality may validate and normalize shared identities [60,61], playing an important role for self-acceptance and well-being, particularly in the face of societal stigma [62], such as exists for SUDs. On this account, universality expressions are likely to be positively associated with recovery outcomes.

Thus, the benefits of peer-to-peer communication for recovery can reflect several communicative processes: an expression of emotional and informational support as well as the sharing of emotions, insights, gratitude, and a sense of connection with others. To understand at a nuanced level how these dynamics relate to recovery benefits, this study assessed their relationship with 3 outcomes: substance use, mood, and quality of life. Substance use directly captures behavior change, but mood and quality of life provide a broad view of day-to-day well-being, with improved mood and quality of life potentially supporting long-term sustainment of recovery [63].

Guided by the prior literature, this study examined the relationships between the forms of expression and recovery outcomes. Building on past work [10-13], we first performed a manual content analysis to identify and code the dominant forms of social support and self-disclosure expressed in the forum and then used the manually coded data to train a series of supervised ML algorithms that we applied to the remaining uncoded data. We then assessed the relationships between 7 expression types and 3 recovery indicators: substance use, mood, and quality of life. Overall, the objective of this study was to (1) deepen our understanding of the forms of peer-to-peer communication that underlie the recovery process, (2) demonstrate a method of generating scalable insights about patients’ status and needs, and (3) inform adaptive interventions for recovery support.

## Methods

### Data Collection and Recruitment

**Figure 1:**
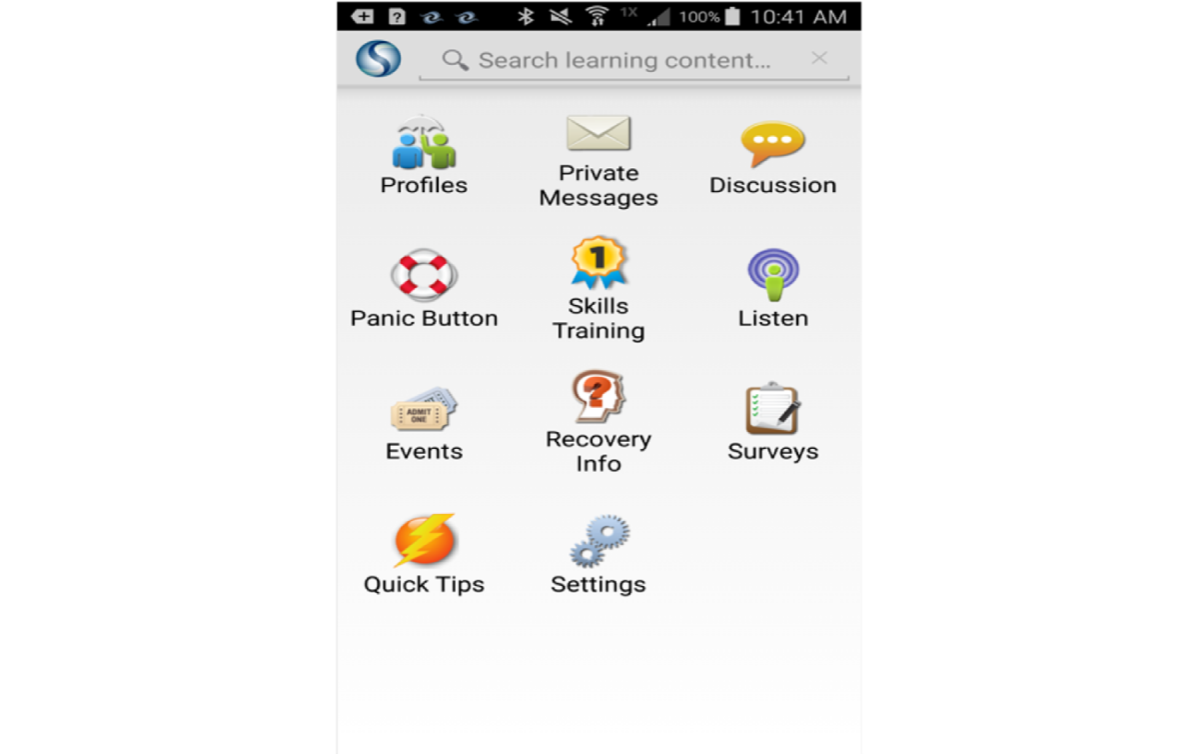
Home page of the Seva app.

Participants were recruited by primary care providers from 3 federally qualified health centers (FQHCs) in the United States in 2014 and offered access to a smartphone-based health app called *Seva* that was designed to connect patients with SUDs to web-based peers as well as offer other informational and communication resources. In a clinical trial, an earlier version of this app reduced the likelihood of risky drinking by half [6]. The app offers various functions, including motivational journal writing, private messaging, games and relaxation activities, meeting and event directories, and the peer-to-peer discussion forum (Figure 1). The contents of the app, including the forum discussions, are in English.

This study draws on text data from these peer-to-peer discussion forums. Specifically, the *discussion* forum is displayed alongside other features that the user can access on demand through the home page. Within this feature, users may select *groups*, including one that represents all of Seva, as well as site-specific groups that are limited to those enrolled at each of the 3 study sites. Within each group, conversations are listed in chronological order, with the most recent first. Users may click on a conversation to see the initial post as well as all replies it received; thus, responses are nested under an original post. Users responding to an existing post trigger a notification to the original poster. Users are all identified to one another by a pseudonym, and the content of the discussions is only available to the research team and participants in the study. In contrast to the discussions that are visible to those within the same site or everyone on the study (depending on the group), Seva also facilitates private messaging among a dyad, as well as personal journaling features. Although not considered here, these communication features have been examined in other research [64].

Participants were recruited by primary care providers from 3 FQHCs in the United States in 2014. Clinicians identified their potentially eligible patients aged >18 years who met the criteria for a SUD diagnosis according to the Diagnostic and Statistical Manual of Mental Disorders, Fourth Edition [65], who they thought may benefit from the study and who had no current psychotic disorder severe enough to prevent participation and no acute medical problem requiring immediate inpatient treatment. After providing informed consent in English, participants received training on how to use Seva, including its discussion forum. The participants also received compensation in the form of payment for the mobile phones and data plans that allow the operation of Seva. A maximum of 100 patients per clinic were recruited through this approach. More details about the app and study design are provided elsewhere [65,66]. To protect participants’ privacy, app use data, including the content of messages posted to the peer-to-peer discussion forum, were stored in a secure encrypted database accessible only to the researchers. The information shared by the participants was anonymized before analysis.

In total, 268 patients were included in the study. Over 6 months, 86.2% (231/268) of these participants posted 10,548 messages on the peer-to-peer forum. We focus here on 93.5% (216/231) of these participants who posted at least 1 message, including a form of social support or self-disclosure. Those who did not post within these categories typically posted only 1 or 2 introductory or greeting messages. Two waves of survey data were collected, capturing demographic characteristics and study outcomes: one at the baseline (before accessing the app) and one after 6 months of using the app. Of the 216 patients in this analysis, all completed the baseline survey, and 180 (83.3%) completed the 6-month survey.

### Ethics Approval

The study received approval from the Health Sciences Institutional Review Board at the University of Wisconsin–Madison (2012-0937-CP020) and has been registered at ClinicalTrials.gov (NCT01963234).

### Informed Consent

All participants provided informed consent, which allows secondary analysis.

### Measurement

#### Human Content Coding

To support our analyses, we first developed a codebook to capture the major types of messages on the forum that may play a role in the recovery process. The codebook was developed from data from an earlier version of the system used in the clinical trial [18,31] and reflected an iterative approach integrating both deductive and inductive techniques [67]. First, we reviewed the literature on peer-to-peer support to deductively define expression types that are central to peer-to-peer communication. As described previously in the literature review, informational and emotional support are well established within peer-to-peer forums. This study adopted coding definitions based on the study by Cohen and Wills [68], renaming *cognitive support* as *informational support*. We developed additional coding categories inductively, based on 3 coders independently reviewing a subset of the messages to observe forms of self-disclosure that emerged in peer-to-peer discussions. On the basis of the codes identified within the data, we conducted a targeted literature review to guide decisions of how to formalize these as coding categories (eg, those literature guided us to code expressions of negative affect and gratitude). These codes were consolidated within a preliminary shared codebook.

Across several rounds of coding, 3 coders overlapped in coding subsets of the data and then met to discuss and adjudicate coding discrepancies and to refine the codebook to better encompass the data, resulting in a formalized codebook for which the coders achieved satisfactory interrater reliability (IRR) for each code (Cohen κ≥0.70) in an overlapping subset of 100 messages per code, consistent with the minimum threshold for computing IRR in prior literature [69]. Coding discrepancies in the overlapping subsets were adjudicated by consensus. Given the substantial effort involved in manual coding, after calculating IRR, the remaining uncoded messages were divided evenly among the coders. Table 1 displays the operationalization of the 7 communication types, along with example messages and the IRR achieved by the coders. Codes were not mutually exclusive, meaning that the same message may include multiple expression types. As Seva is a research forum that is not accessible to the public, only research team members can search the text of messages. Some of the quotes presented in the example message column have been paraphrased.

**Table 1:** Operationalization, example messages, and interrater reliabilities for the 7 expression types.

Expression type	Operationalization	Example message	Cohen κ (human coding)
Emotional support	Messages that foster feelings of comfort and lead the recipient to believe that they are understood, admired, respected, and loved and that others are available to provide caring and security; the writer may convey that they feel for the recipient and wish, or offer prayers and encouragement, for their well-being	“That’s awesome you’re feeling better” [Participant 1]	0.73
Informational support	Messages that relay information, knowledge, and advice to help the receiver understand their world and to adjust to changes within it; the writer may convey how they think it would be useful or appropriate to think about, or respond to, a given situation or may suggest resources, including websites, features of the smartphone app, or other sources	“Exercise helps for anxiety” [Participant 3]	0.78
Negative affect	Messages that reveal the writer’s negative emotions toward events and experiences (eg, fear, anxiety, sadness, anger, or guilt)	“Sometimes I feel like I’m going crazy. Just needed to vent” [Participant 12]	0.84
Change talk	Messages that make an argument for personal behavior change, including conveying ability, need, desire, or reasons to change; commitment to change; and referencing actions linked to behavior change intentions	“I have to put down and stop alcohol and drugs” [Participant 15]	0.70
Insightful disclosure	Messages suggesting that the writer has gained a better or deeper understanding of their experiences over time	“I realized how important it is to take care of myself” [Participant 22]	0.73
Gratitude	Messages giving thanks to specific others or expressing general appreciation	“thanks [username]. [smiley emoji]” [Participant 25]	0.94
Universality	Messages that express recognition of first-hand experience of similar situations or feelings as the recipient	“I too have felt the same, depressed on so many different levels” [Participant 34]	0.72

#### ML Approach

As manual coding for the total 14,393 messages collected in the forum is labor-intensive and time-consuming, this study improved the efficiency of the coding process by applying an ML approach. The 3 coders applied the final codebook to the full set of training data that would be used to develop the ML classifiers. The full codebook was first applied to all 2590 messages exchanged by patients with SUDs within the prior iteration of the app within which the codebook was developed [18,31]. Using this human-coded data as training data, we developed a series of machine classifiers to predict the presence or absence in each message of the aforementioned 7 expression types (emotional support, informational support, negative affect, change talk, insightful disclosure, gratitude, and universality). We iteratively tested algorithm performance, examining *F*_1_-scores to assess when additional manual coding was needed to expand the training set and improve algorithm performance, based on a minimum acceptable threshold of 0.55 based on the *F*_1_-scores provided in prior literature [70-74]. Of the total 14,393 messages that participants exchanged in the study data set (from the deployment within FQHCs), we coded up to 3515 (24.42%) additional messages. Of the 14,393 messages, the total number of human-coded messages ranged from 4467 (31.04%; for negative affect and insightful disclosure) to 6105 (42.42%; for emotional support, informational support, gratitude, and universality). This selective coding process allowed us to reduce the burden on the human coders, leveraging their effort where training data were most needed to improve performance. To assess the ML algorithms, we prepared 1 training set and 4 test sets for each of the 7 expression types, all derived from the human-coded message data set. The training set was drawn from the corpus of coded messages from the study data set that was posted across the entire observation window and was randomly selected to reduce the likelihood of biases owing to changes in the conversation dynamics over time. The human-coded labels were compared with the labels predicted by the algorithms, generating classifier performance statistics across the test sets.

Specifically, drawing on prior work [73], our supervised ML process drew on the linguistic features of messages extracted using the Linguistic Inquiry and Word Count (LIWC) system, which automatically computes the rates at which messages include 90 linguistic features (eg, mentions of *love*, *nice*, and *sweet* are coded as instances of *positive emotion*) [75]. These 90 features were extracted from all training data and test data, and the labels of the training data were used to predict the labels of the test data. Using *scikit-learn* in Python (Python Software Foundation) [76], we used boosted decision tree algorithms—a type of supervised ML where each internal node represents a *test* on an attribute, and each branch represents the outcome of that test, with the performance boosted by making each *tree* dependent on prior trees by fitting the residual of the trees that preceded it. This approach has performed well in similar data sets [72,73,77]. For content categories with imbalanced classes (eg, far more messages did not mention gratitude than those that did), we compensated for the imbalance by oversampling from the minority class using the Synthetic Minority Oversampling Technique [78]. Specifically, after splitting the data set into training and testing sets, we generated synthetic samples from the minority class to extend the training data set. These synthetic samples are created to closely resemble but not exactly replicate existing samples within the minority class. The other Python packages used in the study are *pandas* [79], *NumPy* [80], *pickle* [81], and *Natural Language Toolkit* [82].

#### Survey Measures

Substance use (ie, any drinking or drug use) and well-being outcomes (ie, positive mood and quality of life) were self-reported through baseline and 6-month surveys.

##### Any Substance Use

At both baseline and the 6-month follow-up, the survey asked participants to report whether they had consumed any alcoholic beverages in the past 30 days. In addition, the survey asked whether participants had used any illegal or street drugs or abused any prescription medications in the past 30 days. To capture whether participants engaged in any substance use, we created a variable combining these 2 binary measurements, that is, recoding as *Yes* if they engaged either in drinking or drug use.

##### Mood

The Positive and Negative Affect Schedule–Short Form (PANAS-SF) was used to measure mood [83]. The scale consists of 10 items, of which 5 measure positive affect, and 5 measure negative affect. Cronbach α values for the positive affect and negative affect dimensions were .83 and .80, respectively. This study adopts a single dimension by reversing the negative items (Cronbach α=.80), following Kim and Hatfield [84] and Furlong et al [85], producing a measure of mood as a single dimension. We also constructed positive and negative affect subscales so that we could test these components.

##### Quality of Life

The Patient-Reported Outcomes Measurement Information System (PROMIS) Global-10 measure consists of 10 items assessing general functioning and well-being across physical and mental health domains [86]. PROMIS assesses the general health care–related quality of life. Consistent with the PROMIS scoring guideline that combines the 2 dimensions of health [86], 3 of the 10 items were recoded such that all items were scored on a scale from 1 to 5, with higher scores indicating higher functioning, after which all 10 items were averaged (Cronbach α=.73). We also estimated reliability for the physical and mental health subscales (Cronbach α for physical health=.68 and Cronbach α for mental health=.81).

The zero-order correlations for the 7 expression types were also assessed, as presented in Table 2. The correlations ranged from −0.24 (emotional support and negative affect) to 0.54 (insightful disclosure with both change talk and universality), with all falling below the threshold of 0.80 that might raise concerns about multicollinearity if entered simultaneously in regression models [87].

**Table 2:** Zero-order correlations for the 7 expression types (n=216)^a^.

	Emotional support	Informational support	Negative affect	Change talk	Insightful disclosure	Gratitude	Universality disclosure
Emotional support	1	0.3	−0.24	−0.19	−0.21	−0.10	−0.09
Informational support	0.3	1	0.07	0.08	0.2	−0.20	0.27
Negative affect	−0.24	0.07	1	0.3	0.41	0.15	0.35
Change talk	−0.19	0.08	0.3	1	0.54	0.32	0.27
Insightful disclosure	−0.21	0.2	0.41	0.54	1	0.31	0.54
Gratitude	−0.10	−0.20	0.15	0.32	0.31	1	0.18
Universality	−0.09	0.27	0.35	0.27	0.54	0.18	1

^a^We also calculated the mean and SD of the 7 types of expression: emotional support (mean 0.41, SD 0.23), informational support (mean 0.23, SD 0.20), negative affect (mean 0.07, SD 0.10), change talk (mean 0.07, SD 0.11), insightful disclosure (mean 0.16, SD 0.19), gratitude (mean 0.27, SD 0.20), and universality disclosure (mean 0.08, SD 0.10).

### Statistical Analysis

The 2 waves of survey data and the machine-coded discussion forum data were merged based on the participants’ unique identifiers. For the discussion forum data, the count of messages falling within each expression category was aggregated for each user over the 6-month study period. As enrollment was conducted on a rolling basis, time on the study was relative to each participant’s start date. Given that the raw counts of messages in each category can be biased by the participants’ uneven message production (ie, some participants produce many more messages than others), we created a proportion score for each expression type, dividing the count of messages within each expression type by the total number of messages posted by the participant. Therefore, the value of the emotional support measure ranges from 0 to 1 (a 1-unit change in this measure). Hierarchical ordinary least squares (OLS) and logistic regression models were used to evaluate the relationships between the proportion of an individual’s messages in each category and the 3 recovery outcomes as assessed at 6 months, controlling for the baseline value of the outcome. All models also controlled for baseline alcohol and other drug use, age, sex, education, and race because they were significant predictors of SUD recovery in prior research [88,89]. SPSS software (version 25.0; IBM Corp) was used for the regression analyses.

## Results

### Participant and Message Characteristics

Table 3 summarizes participants’ demographics and outcome measurements from the 2 waves. Despite some attrition of participants (36/216, 16.7%) between the baseline and 6-month surveys, we did not find demographic differences between those who remained on the study and those who did not.

**Figure 2:**
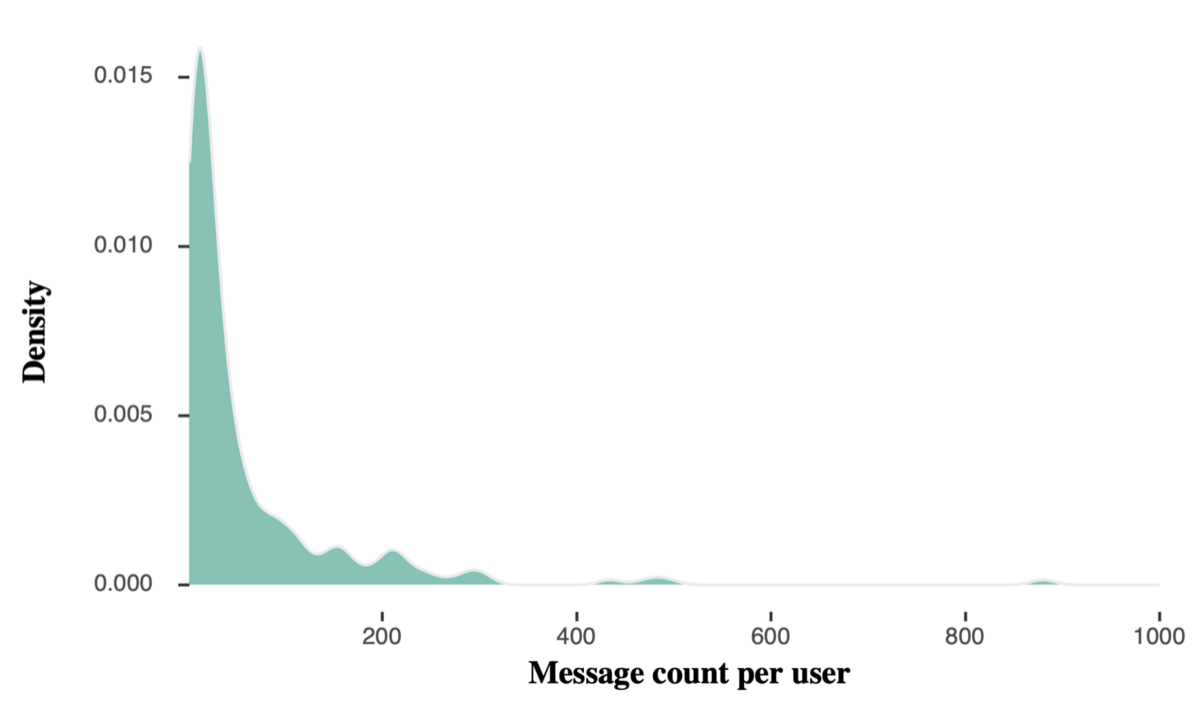
Distribution of message count by Seva app user.

A total of 10,503 messages were posted by the participants (n=216) over 6 months, most frequently in the first 2 months. The average length of messages in the study data set was 146.37 (SD 75.00) characters, with the median length being 139.00 (IQR 31.00-170.00). Of the 10,503 messages, 1576 (15.01%) were original messages (starting a new thread), with the rest being replies to an existing thread. The users each produced on average 72 (SD 164) messages over 6 months on the study. Figure 2 displays the density distribution of message numbers per user. The density is derived on Gaussian kernels from the R graphing package *ggplot*.

**Table 3:** Descriptive statistics of participants at baseline and 6-month surveys.

Demographics		Baseline (n=216)	6-month survey (n=180)
**Education, n (%)**			
	Less than college	108 (50)	87 (48.3)
	Some college	64 (29.6)	57 (31.7)
	More than college	44 (20.3)	36 (20)
**Sex, n (%)**			
	Male	114 (52.8)	94 (52.2)
	Female	102 (47.2)	86 (47.8)
**Race, n (%)**			
	People of color	73 (33.8)	60 (33.3)
	White	143 (66.2)	120 (66.7)
Age (years), mean (SD)		41.8 (10.60)	42.3 (10.73)
**Outcomes**			
	Any drinking (yes), n (%)	68 (31.5)	40 (18.5)
	Drug use (yes), n (%)	60 (27.8)	27 (12.5)
	Positive mood, mean (SD)	3.22 (0.76)	3.54 (0.71)
	Quality of life, mean (SD)	28.48 (9.70)	30.02 (7.02)

### ML Performance

Table 4 presents the final algorithm performance indexes for each expression type, as averaged across the 4 test sets. The boosted decision tree ML algorithms achieved *F*_1_-scores ranging from 0.57 to 0.85, area under the receiver operating characteristic curve values ranging from 0.72 to 0.86, sensitivity scores ranging from 0.75 to 0.95, and specificity scores ranging from 0.65 to 0.79.

On the basis of the machine classifiers applied to the entire study data set, emotional support was the most common expression type (4788/10,503, 45.59%), followed by gratitude (2972/10,503, 28.3%), informational support (2514/10,503, 23.94%), insightful disclosure (1514/10,503, 14.41%), universality (901/10,503, 8.58%), negative affect (731/10,503, 6.96%), and change talk (707/10,503, 6.73%). Table 5 displays the average proportion of messages in each category in each study month for each expression type. In linear growth curve models assessing change in monthly expression in each category (not shown), only 2 expression types had slopes significantly different from 0: emotional support (β=.012; *P*=.03) and universality (β=.008; *P*=.007), with both increasing over time.

**Table 4:** F_1_-scores, area under the receiver operating characteristic curve (AUROC) values, and sensitivity and specificity scores for boosted decision tree algorithm performance for the 7 expression types.

Expression types	*F*_1_-score	AUROC	Sensitivity	Specificity
Emotional support	0.65	0.72	0.75	0.70
Informational support	0.71	0.76	0.79	0.72
Negative affect	0.57	0.79	0.86	0.73
Change talk	0.85	0.86	0.95	0.74
Insightful disclosure	0.60	0.76	0.86	0.65
Gratitude	0.70	0.78	0.81	0.79
Universality	0.59	0.79	0.83	0.75

**Table 5:** Proportion of messages posted within each of the 7 expression categories by study month.

	Month 1 (n=216), average proportion (SD)	Month 2 (n=190), average proportion (SD)	Month 3 (n=163), average proportion (SD)	Month 4 (n=136), average proportion (SD)	Month 5 (n=115), average proportion (SD)	Month 6 (n=97), average proportion (SD)
Emotional support	0.40 (0.31)	0.40 (0.32)	0.47 (0.34)	0.45 (0.35)	0.48 (0.33)	0.44 (0.33)
Informational support	0.20 (0.26)	0.25 (0.28)	0.22 (0.26)	0.25 (0.28)	0.25 (0.29)	0.26 (0.30)
Negative affect	0.06 (0.12)	0.09 (0.17)	0.08 (0.18)	0.08 (0.17)	0.11 (0.22)	0.07 (0.18)
Change talk	0.07 (0.17)	0.07 (0.17)	0.06 (0.16)	0.08 (0.17)	0.11 (0.20)	0.08 (0.18)
Insightful disclosure	0.14 (0.21)	0.18 (0.27)	0.19 (0.26)	0.18 (0.24)	0.18 (0.27)	0.15 (0.21)
Gratitude	0.26 (0.24)	0.25 (0.25)	0.27 (0.29)	0.28 (0.31)	0.27 (0.28)	0.25 (0.29)
Universality	0.06 (0.12)	0.09 (0.18)	0.07 (0.13)	0.09 (0.17)	0.12 (0.22)	0.11 (0.22)

### Regression Analysis

A series of hierarchical OLS and logistic regression models were run to predict substance use, mood, and quality of life at 6 months based on the 7 types of expression within the peer-to-peer discussion forum. We controlled for the baseline measurement of each outcome, baseline drinking and drug use, and demographic covariates as described previously. For substance use, Table 6 shows that expressing a higher proportion of emotional support was significantly (*P*=.03) associated with a decreased likelihood of substance use. After controlling for baseline substance use, a 1-unit increase in emotional support proportion is associated with an 88% decrease in the odds of reporting substance use at 6 months (odds ratio 0.12, CI 0.016-0.83; *P*=.03). The standardized coefficient estimates suggest that compared with the other 6 types of expression, emotional support had the strongest negative association with the change of any drinking or drug use at 6 months (standardized β=−.49; *P*=.03).

For well-being, we found that expressing a greater proportion of messages containing negative affect was significantly associated with lower positive mood score at 6 months (Table 7), with a 1-unit increase in the proportion of negative affect expression within a message being associated with a 1.49-point decline in the mood score at 6 months as measured on the PANAS-SF compared with baseline (β=−1.49; *P*=.007). Considering the typical use of the PANAS-SF as 2 dimensions (ie, positive mood and negative mood), we also applied OLS regression to the positive and negative subscales separately. We found a significant negative relationship between negative affect expression and positive mood (*P*=.02), whereas the relationship between negative affect expression and negative mood did not achieve significance (*P*=.09). These results are presented in Multimedia Appendix 1. This model, including expression types and baseline outcome control as the predictors, explained 62% of the variance in mood change (*F*_13,164_=7.73; *P*<.001; *R*^2^=0.62). Similarly, greater negative affect expression was significantly associated with lower reported quality of life at 6 months compared with baseline, with a 1-unit increase in the proportion of negative affect expressions being associated with an 11-point reduction on the PROMIS scale (β=−11.00; *P*=.05; Table 8). Conversely, the proportion of universality expressions was found to be positively associated with quality of life (β=11.83; *P*=.04), with a 1-unit increase associated with an 11-point increase on the PROMIS scale compared with baseline. The model fit the data well (*F*_13,160_=7.64; *P*<.001; *R*^2^=0.62).

In summary, we found significant relationships between recovery outcomes and a subset of the expression types examined. The proportion of emotional support expression was positively associated with a reduced likelihood of substance use but not well-being outcomes at 6 months. We did not find any significant relationships between the proportion of informational support expressions and recovery outcomes. The proportion of negative affect expressions was not significantly related to substance use but was linked with decreased mood and quality of life. We found no significant relationships between recovery outcomes and the proportion of expressions that included change talk, insightful disclosure, or gratitude. Finally, the proportion of universality expressions was associated with positive mood but not substance use or quality of life.

**Table 6:** Hierarchical logistic regression models using expression types to predict whether individuals engage in any drinking or drug use at 6 months.

			Model 1^a^									Model 2^b^						
			Unstandardized β estimates		Standardized β estimates		OR^c^ (95% CI)		*P* value		Unstandardized β estimates			Standardized β estimates		OR (95% CI)		*P* value
**Demographics**																		
	Age	−.020		−.240		0.97 (0.94-1.01)		.19		−.020			−.230		0.98 (0.94-1.10)		.22	
	Education	−.100		−.050		0.91 (0.44-1.88)		.80		−.100			−.050		0.90 (0.43-1.90)		.79	
	Sex: male	−.007		.000		0.99 (0.48-2.04)		.99		−.090			−.040		0.92 (0.43-1.97)		.82	
	Race: White	.170		.080		1.19 (0.54-2.59)		.67		.050			.020		1.05 (0.45-2.46)		.92	
	Baseline drinking and drug use	1.720		.860		5.56 (2.65-11.68)		<.001		1.820			.090		6.14 (2.84-13.26)		<.001	
**Expression types**																		
	Emotional support	N/A^d^		N/A		N/A		N/A		−2.150			−.490		0.12 (0.016-0.83)		.03	
	Informational support	N/A		N/A		N/A		N/A		1.740			.350		5.70 (0.56-0.44)		.79	
	Negative affect	N/A		N/A		N/A		N/A		−.690			−.070		0.50 (0.00-58.066)		.75	
	Change talk	N/A		N/A		N/A		N/A		−.530			−.060		0.59 (0.01-42.24)		.81	
	Insightful disclosure	N/A		N/A		N/A		N/A		.060			.010		1.06 (0.07-15.33)		.96	
	Gratitude	N/A		N/A		N/A		N/A		.750			.140		2.12 (0.17-26.26)		.56	
	Universality	N/A		N/A		N/A		N/A		−.590			−.060		0.56 (0.01-45.08)		.79	

^a^The intercept for model 1 is −0.90 (log likelihood=187.81, Cox & Snell *R*^2^=0.15, and McFadden *R*^2^=0.14).

^b^The intercept for model 2 is −0.44 (log likelihood=151,67, Cox & Snell *R*^2^=0.18, and McFadden *R*^2^=0.17).

^c^OR: odds ratio.

^d^N/A: not applicable.

**Table 7:** Hierarchical linear regression models using expression types to predict positive mood at 6 months^a^.

			Model 1^b^							Model 2^c^							Model 3^d^				
			Unstandardized β estimates		Standardized β estimates		*P* value		Unstandardized β estimates			Standardized β estimates		*P* value		Unstandardized β estimates			Standardized β estimates		*P* value
**Demographics**																					
	Age	.002		.030		.73		−.007			−.100		.13		−.008			−.120		.08	
	Education	−.090		.110		.40		−.080			−.060		.35		−.060			−.040		.50	
	Sex: male	.180		.110		.11		.140			.100		.12		.120			.090		.19	
	Race: White	−.060		.120		.60		−.080			−.050		.40		−.070			−.050		.48	
Baseline positive mood			N/A^e^		N/A		N/A		.580			N/A		<.001		.580			.610		<.001
Baseline drinking and drug use			N/A		N/A		N/A		.100			N/A		.29		.120			.080		.22
**Expression types**																					
	Emotional support	N/A		N/A		N/A		N/A			N/A		N/A		−.150			−.050		.54	
	Informational support	N/A		N/A		N/A		N/A			N/A		N/A		−.100			−.030		.72	
	Negative affect	N/A		N/A		N/A		N/A			N/A		N/A		−1.490			−.210		<.001	
	Change talk	N/A		N/A		N/A		N/A			N/A		N/A		.840			.110		.12	
	Insightful disclosure	N/A		N/A		N/A		N/A			N/A		N/A		.220			.060		.51	
	Gratitude	N/A		N/A		N/A		N/A			N/A		N/A		.020			.005		.95	
	Universality	N/A		N/A		N/A		N/A			N/A		N/A		−.180			−.030		.75	

^a^*Male, White*, and *baseline any drinking and drug use* were dummy coded as *Male*=1, *White*=1, and *Had any drinking and drug use*=1. *Education* is an ordinal scale.

^b^The intercept for model 1 is 3.46 (*R*^2^=0.16; *F*^2^=0.19).

^c^The intercept for model 2 is 1.96 (*R*^2^=0.59; *F*^2^=1.44).

^d^The intercept for model 3 is 2.09 (*R*^2^=0.62; *F*^2^=1.63).

^e^N/A: not applicable.

**Table 8:** Hierarchical linear regression models using expression types to predict quality of life (QOL) at 6 months^a^.

			Model 1^b^							Model 2^c^							Model 3^d^				
			Unstandardized β estimates		Standardized β estimates		*P* value		Unstandardized β estimates			Standardized β estimates		*P* value		Unstandardized β estimates			Standardized β estimates		*P* value
**Demographics**																					
	Age	−.13		−.19		.01		−.11			−.17		.01		−.13			−.19		.006	
	Education	1.21		.09		.26		.97			.07		.29		1.40			.10		.13	
	Sex: male	1.67		.12		.12		1.50			.11		.10		1.53			.11		.11	
	Race: White	1.49		.10		.19		1.25			.08		.20		1.32			.09		.20	
Baseline QOL			N/A^e^		N/A		N/A		.58			.52		<.001		.58			.53		<.001
Baseline drinking and drug use			N/A		N/A		N/A		−.23			−.02		.81		−.27			−.02		.78
**Expression types**																					
	Emotional support	N/A		N/A		N/A		N/A			N/A		N/A		.37			.01		.88	
	Informational support	N/A		N/A		N/A		N/A			N/A		N/A		−1.99			−.06		.48	
	Negative affect	N/A		N/A		N/A		N/A			N/A		N/A		−11.00			−.16		.05	
	Change talk	N/A		N/A		N/A		N/A			N/A		N/A		−2.04			−.03		.71	
	Insightful disclosure	N/A		N/A		N/A		N/A			N/A		N/A		−1.54			−.04		.65	
	Gratitude	N/A		N/A		N/A		N/A			N/A		N/A		−1.02			−.03		.73	
	Universality	N/A		N/A		N/A		N/A			N/A		N/A		11.83			.17		.04	

^a^*Male*, *White*, and *baseline any drinking and drug use* were dummy coded as *Male*=1, *White*=1, and *Had any drinking and drug use*=1. *Education* is an ordinal scale.

^b^The intercept for model 1 is 32.99 (*R*^2^=0.26; *F*^2^=0.35).

^c^The intercept for model 2 is 16.28 (*R*^2^=0.59; *F*^2^=1.44).

^d^The intercept for model 3 is 17.31 (*R*^2^=0.62; *F*^2^=1.63).

^e^N/A: not applicable.

## Discussion

### Principal Findings

Peer-to-peer communication within SUD apps can provide a wealth of data about individuals’ experiences and likely health trajectories, but a review by hand and the coding of messages can quickly become prohibitively effortful and time consuming. This study makes a methodological contribution by demonstrating that supervised ML can be applied to perform large-scale automated content analysis in a peer-to-peer discussion group context. The ML performance was consistent with that achieved using similar methods in prior literature [70-74], with variation across content categories ranging from moderate to good performance. In turn, this content analysis allowed us to examine the dominant ways in which individuals engage in app-based peer-to-peer communication over 6 months and to explore how each communication style relates to the user’s recovery course. Our results add to a growing body of evidence suggesting that informal online peer-to-peer conversations provide a valuable source of information about participants’ future health trajectories [12,90] and point to giving emotional support and expressing a sense of universality as potential predictors of reduced substance use and improved quality of life, respectively. By contrast, negative affect expressions were associated with lower quality of life and mood. As discussed in the following paragraphs, these findings deepen our understanding of how peer-to-peer communication may bolster recovery as well as suggest potential opportunities to improve how app-based SUD interventions adapt to better meet users’ needs.

Our findings add to a growing body of work suggesting that the expression of social support, particularly emotional support, is a dominant expression type of peer-to-peer forums and can potentially benefit individuals in their recovery. Of course, because these data are correlational, the reverse is also possible, meaning that those experiencing positive recovery outcomes express themselves in particular ways. Expressing emotional support was negatively associated with substance use after 6 months of accessing the app, which is consistent with other work in the domain of SUD recovery [91]. Indeed, this was the only communication type that was associated with this behavioral outcome. We did not find evidence of a similar role for informational support, which could reflect that informational support can be more challenging or potentially burdensome to give than emotional support [31,92]. These findings suggest a need to distinguish between giving informational support and giving emotional support when examining social support in peer-to-peer communication.

Our research also looks beyond social support to consider self-disclosure. Although less often examined than social support in peer-to-peer forums, self-disclosure can be an important method of soliciting support and can provide a pathway to better understand one’s own recovery process or to connect to other group members through finding areas of shared experience and understanding. Whereas some past literature has proposed potential benefits that come from openly expressing negative emotions, our findings are more consistent with those of prior research, which found that high levels of self-disclosure of negative affect predict health risks [93], likely capturing individuals’ underlying struggles and challenges. Finally, although past work has noted that experiences of oneness or universality are common in peer support groups [60], this work establishes that individual differences in expressing such feelings distinguish those more likely to have greater well-being at follow-up. Experiences of universality and belonging may be important in the SUD context to counteract the feelings of alienation and loneliness that individuals frequently experience [57,59].

Our analysis of the monthly proportions of message types shows that both emotional support and universality expression tended to increase as a proportion of total messages as the patients engaged with this support forum over time. The patterns of increasing emotional support are consistent with prior work suggesting that participants in support groups often move from primarily receiving to giving support as their health trajectories become more stable, and they feel more confident with their role in the group [94]. Longer-term participation may also allow for finding commonalities and building a sense of belonging, as reflected in an increasing proportion of universality expressions.

These findings have implications for the design of online support forums. First, although our findings are correlational, it may be worth considering whether designers can make support forums more conducive to certain communication styles that are associated with improvements in recovery outcomes; for example, our findings diverge for emotional support giving and informational support giving, which may warrant a greater focus on buttressing emotional support giving. Systems could attempt to address this by offering guidance to support givers, perhaps encouraging emotional support delivery or giving training for those who do want to give informational support so that they can also find applications to their own recovery process. For universality, structured activities may support finding commonalities with others. Alternatively, the perceptions of similarity may be increased by matching group participants based on similarities or by displaying information about other participants that highlights shared experience [37].

Our findings also show that the expression types linked to positive recovery outcomes (ie, emotional support giving and universality expressions) increased over time as a proportion of messages sent, suggesting the potential importance of sustaining participation and motivating more participants to interact. Often, as in the case of any online discussion forum, peer-to-peer forums face a *cold start problem*, wherein early contributions to a forum generally receive little engagement; yet, without engagement, other participants are unlikely to participate [95]. This has led to strategies such as seeding a group with highly motivated participants to create a sense of community, given that participation is based on anticipated future interaction from group members [35,96]. Studies also suggest tailoring the engagement tactics based on a forum’s life cycle [97], including investing time early to engage core members [98].

Real-time understanding of communication dynamics may also facilitate just-in-time intervention and system adaptation; for example, expressing a high proportion of messages that convey negative affect may be an important signal that reflects when participants are struggling and need additional support. This support might come from peers (eg, by prompting peers to provide support to a specific participant), professionals (eg, system *moderators* may reach out privately or respond to threads involving this participant), or from automated system features (eg, a chatbot may launch a dialogue, or the system might recommend or launch modules to cope with negative emotions). Similarly, individuals who participate without expressing emotional social support could potentially benefit from tailored guidance in delivering this type of support, whereas such guidance may not be indicated for others.

Finally, this study does not develop or test models that predict real-time relapse, but the communication types examined here could be considered as potential features to integrate into such models, allowing for identifying specific moments when individuals may need support and resources. A growing body of work shows that peer-to-peer communication dynamics (eg, communication on social media) are associated with individuals’ health behaviors [90]. Integrating algorithmically captured content categories can potentially improve upon past approaches because we focused on communication types that are prevalent in peer-to-peer communication for SUDs and where the literature suggests potential relationships to an individual’s recovery course. The rates of producing content falling into these categories can be computed automatically, essentially in real time. Although most of the communication types examined were not related to substance use at 6 months, future research can look at both communication and health behavior at a more granular timescale. Past work suggests that models that incorporate heterogeneous features often perform best in real-time prediction [10], and thus peer-to-peer communication dynamics may be considered alongside other features such as app use, location, movement, and ecological momentary assessment.

This study has limitations. First, we examined only the effects of giving support and not receiving it. Receiving support of various forms is also associated with individuals’ recovery outcomes [31], and the types of support received could potentially be automatically assessed using similar methods. A second limitation is that our measurement of substance use did not encompass recovery efforts other than abstinence, such as by capturing reduction in use. According to a recent recommendation [99], recovery outcomes should reflect 3 components: biopsychosocial functioning, remission, and cessation. Moreover, our sample may not represent those with SUDs as a whole because the participants were drawn from among those in contact with the primary care system. These participants were also willing to consent to the study, and they engaged with the app by voluntarily communicating via the support forum. It is unclear whether the same patterns of communication and associations with recovery outcomes would emerge in other populations with SUDs or if tested using social networking platforms (eg, Facebook) or messaging tools (eg, texting or SMS text messaging). We are also unable to use these methods for those participants who never post, and the utility may be reduced for those who post infrequently. There may be expression types that are more strongly associated with recovery and well-being outcomes for those who post more versus those who post less within the forum. It is also likely that associations between expression and health and well-being outcomes are more robust among individuals who post more often. As with any manual content analysis, it is possible that our codebook may have been biased toward emphasizing certain patterns of communication and overlooking other important dynamics. As with any study of supervised ML, it is also possible that the automated coding process may be biased to systematically overlook or misclassify some patterns of language. Future work may apply innovations in ML such as neural networks to improve the classifiers [100]. In addition, more robust classification approaches would include multifold cross-validation [101] and a holdout validation set to assess classifier performance in new data, reduce bias in the classification models, and facilitate transfer learning [102]. Furthermore, messages from the same participants may have been in both the training and test sets, whereas a more conservative approach would be to have mutually exclusive sets of participants in the training and test sets.

A final limitation is that the association between communication styles and recovery outcomes is correlational. We cannot say with confidence whether the relationships observed are causal (ie, whether communicating in a particular way produces changes in the recovery course). However, to increase the strength of the claims we can make, we attempted to control for potential confounds. Furthermore, the data overwhelmingly come from the first several months on the study, with these expressions used to predict outcomes months later such that temporal ordering is consistent with a potentially causal relationship.

### Conclusions

With the prevalence of the internet, online peer-to-peer support forums represent a growing support venue for those in recovery. This study extracted 7 types of messages exchanged on a smartphone-based forum and applied supervised ML to perform large-scale quantitative content analysis over 6 months. Analyses leveraging the machine-coded data suggest forms of peer-to-peer communication that distinguish individuals’ likely recovery course, notably emotional support, universality, and negative affect expressions. Attending to these forms of expression may help to develop interventions that better respond to participants’ recovery needs.

## Appendix

Multimedia Appendix 1 Extra regression models on the Positive and Negative Affect Schedule–Short Form.

## Abbreviations

FQHC: federally qualified health center
IRR: interrater reliability
LIWC: Linguistic Inquiry and Word Count
ML: machine learning
OLS: ordinary least squares
PANAS-SF: Positive and Negative Affect Schedule–Short Form
PROMIS: Patient-Reported Outcomes Measurement Information System
SUD: substance use disorder

## Data Availability

We are not able to make the discussion post data from this study publicly available owing to the sensitive nature of the peer-to-peer communication data in recovery support forums and the fact that the participants did not consent to broad sharing of the research data. Nonetheless, numerical data underlying the regression analyses may be provided upon reasonable request to the corresponding author.
