# Supplementary material for: Using Machine Learning of Online Expression to Explain Recovery Trajectories: Content Analytic Approach to Studying a Substance Use Disorder Forum
Source: J Med Internet Res. 2023 Aug 22;25:e45589. doi: 10.2196/45589 (PMC10481212; doi:10.2196/45589)
Supplement: Multimedia Appendix 1 [file jmir_v25i1e45589_app1.docx]

# Appendix

*Table S1*. Hierarchical Linear Regression Models Using Expression Types to Predict the Positive Dimension of PANAS at Six Months.

|  | Model 1 | | | | Model 2 | | | Model 3 | | |
| --- | --- | --- | --- | --- | --- | --- | --- | --- | --- | --- |
|  | Unstandardized Estimates | Standardized Estimates | | *P* value | Unstandardized Estimates | Standardized Estimates | *P* value | Unstandardized Estimates | Standardized Estimates | *P* value |
| Demographic |  | |  |  |  |  |  |  |  |  |
| Age | -0.01 | | -0.10 | 0.21 | -0.01 | -0.14 | 0.04 | -0.01 | -0.17 | 0.02 |
| Education | -0.04 | | -0.02 | 0.79 | -0.09 | -0.05 | 0.49 | -0.06 | -0.03 | 0.64 |
| Male | 0.04 | | 0.02 | 0.78 | 0.02 | 0.01 | 0.90 | -0.03 | -0.02 | 0.83 |
| Caucasian | -0.17 | | -0.09 | 0.26 | -0.12 | -0.06 | 0.36 | -0.15 | -0.08 | 0.28 |
|  |  | |  |  |  |  |  |  |  |  |
| Baseline positive dimension |  | |  |  | 0.50 | 0.51 | <.001 | 0.51 | 0.51 | <.001 |
| Baseline drinking/drug use |  | |  |  | 0.04 | 0.02 | 0.75 | 0.05 | 0.03 | 0.69 |
|  |  | |  |  |  |  |  |  |  |  |
| Expression Types |  | |  |  |  |  |  |  |  |  |
| Emotional support |  | |  |  |  |  |  | -0.11 | -0.03 | 0.73 |
| Informational support |  | |  |  |  |  |  | 0.08 | 0.02 | 0.84 |
| Negative affect |  | |  |  |  |  |  | -1.70 | -0.19 | 0.02 |
| Change talk |  | |  |  |  |  |  | 1.05 | 0.11 | 0.15 |
| Insightful disclosure |  | |  |  |  |  |  | -0.16 | -0.03 | 0.73 |
| Gratitude |  | |  |  |  |  |  | -0.46 | -0.09 | 0.25 |
| Universality |  | |  |  |  |  |  | 0.65 | 0.07 | 0.40 |
| (Intercept) | 3.94 | |  | <.001 | 2.41 |  | <.001 | 2.70 |  | <.001 |
| R^2^ | 0.02 | |  | 0.63 | 0.26 |  | <.001 | 0.30 |  | <.001 |
| F^2^ | 0.02 | |  |  | 0.35 |  |  | 0.43 |  |  |

*Note*. Male, Caucasian and baseline any drinking/drug use were dummy coded as “Male” = 1, “Caucasian” = 1, and “Had any drinking/drug use” = 1. Education is an ordinal scale.

*Table S2*. Hierarchical Linear Regression Models Using Expression Types to Predict the Negative Dimension of PANAS at Six Months.

|  | Model 1 | | | Model 2 | | | Model 3 | | |
| --- | --- | --- | --- | --- | --- | --- | --- | --- | --- |
|  | Unstandardized Estimates | Standardized Estimates | *P* value | Unstandardized Estimates | Standardized Estimates | *P* value | Unstandardized Estimates | Standardized Estimates | *P* value |
| Demographic |  |  |  |  |  |  |  |  |  |
| Age | -0.01 | -0.14 | 0.07 | -0.001 | -0.01 | 0.86 | 0.00 | -0.006 | 0.94 |
| Education | 0.15 | 0.08 | 0.29 | 0.09 | 0.05 | 0.46 | 0.07 | 0.04 | 0.56 |
| Male | -0.31 | -0.17 | 0.02 | -0.28 | -0.15 | 0.02 | -0.27 | -0.15 | 0.03 |
| Caucasian | -0.05 | -0.03 | 0.74 | 0.03 | 0.02 | 0.79 | -0.01 | -0.006 | 0.93 |
|  |  |  |  |  |  |  |  |  |  |
| Baseline negative dimension |  |  |  | 0.47 | 0.48 | <.001 | 0.49 | 0.50 | <.001 |
| Baseline drinking/drug use |  |  |  | -0.09 | -0.05 | 0.44 | -0.12 | -0.07 | 0.33 |
|  |  |  |  |  |  |  |  |  |  |
| Expression Types |  |  |  |  |  |  |  |  |  |
| Emotional support |  |  |  |  |  |  | 0.17 | 0.04 | 0.60 |
| Informational support |  |  |  |  |  |  | 0.19 | 0.04 | 0.61 |
| Negative affect |  |  |  |  |  |  | 1.23 | 0.14 | 0.09 |
| Change talk |  |  |  |  |  |  | -0.58 | -0.06 | 0.42 |
| Insightful disclosure |  |  |  |  |  |  | -0.54 | -0.11 | 0.24 |
| Gratitude |  |  |  |  |  |  | -0.45 | -0.09 | 0.27 |
| Universality |  |  |  |  |  |  | 1.13 | 0.12 | 0.14 |
| (Intercept) | 3.02 |  | <.001 | 1.17 |  | 0.004 | 1.09 |  | 0.02 |
| R^2^ | 0.07 |  | 0.02 | 0.27 |  | <.001 | 0.31 |  | <.001 |
| F^2^ | 0.08 |  |  | 0.37 |  |  | 0.45 |  |  |

*Note*. Male, Caucasian and baseline any drinking/drug use were dummy coded as “Male” = 1, “Caucasian” = 1, and “Had any drinking/drug use” = 1. Education is an ordinal scale
